# Three Cases of Purpura Hæmorrhagica

**Published:** 1885-09

**Authors:** George W. Winterburn

**Affiliations:** New York


### THREE CASES OF PURPURA HÆMORRHAGICA.

George W. Winterburn, M. D., New York.

To begin with an Irishism, neither of these three cases of purpura could be strictly classed under that head. I have never seen a real case of purpura—that is, a case in which the cutaneous extravasation is the principal feature of the case. Of these three cases, one is rheumatic fever, another an intermittent fever, and the third a gastro-enteritis, but they all have as a prominent though secondary condition purpuric extravasations, which were evidently reciprocal, as they yielded, in common with all the other symptoms, to the remedy homœopathic to the case. They were each quite out of the ordinary run of cases and of a severity to cause great anxiety, but they afford beautiful illustrations of the proper way to "take" a case, and of the power of the properly selected dynamized remedy to stay the progress of the most dangerous retrograde metamorphoses, and of the recuperating energy of the human system when so aided.

### I. A Rhus Venenata Case.

James S., aged twenty-nine; of the bilious type, lean and spare but not emaciate; by trade a carpenter, but at present employed on the elevated railway; married; had intermittent fever several years ago, and is somewhat subject to rheumatic attacks; applied at the Manhattan Hospital for treatment October 10th, 1879. He complained of an intense headache, describing the pain as throbbing. He felt dizzy when turning or stooping, but had no nausea. The conjunctiva was reddened and dry, the face somewhat flushed; temp., 100.2; pulse, seventy-eight; respiration, twenty. The pulse was rather hard, and the heart beat with a sharp click. He was given Glonoine $^{12}$ every two hours.

October 11th.—Headache no better. The face more deeply congested; the conjunctiva about as yesterday, but the eyes look more staring. The brain seems to have a wavy, undulating motion whenever he stirs, but especially when stooping. He refuses to take his medicine, as he imagines it disagrees with him, and thinks he has been poisoned. Complains of pain in the left wrist and throbbing in the hand, which seemed to be synchronous with the throbbing in the head. Temp., 100.6; pulse, eighty-two, and of about the same general character, respiration, twenty.

A study of the pathogenesy of Glonoine confirmed the impression that it was the remedy most homœopathic to the case. Glonoine has—

Throbbing: in temples, in vertex, in occiput, in whole head.

Severe pain in the forehead, throbbing in the temples, worse from walking.

Headaches worse: from shaking or jarring the head, stooping, bending it backward, after lying down, when ascending steps, in damp weather, in the sun.

Vertigo worse: from stooping, or moving the head.

Fear: apprehensive of approaching death; fears she has been poisoned.

Face flushed, hot, especially about the eyes and forehead, with headache; livid, purple.

Eyes injected, red, protruding, wild, staring.

Pulse: accelerated, increased during headache; quick, small, irregular.

Weakness of wrists after headache.

Rheumatic pains in fingers of left hand.

Feels pulse in fingers.

Thus assured of the homœopathicity of the remedy, although no improvement had taken place, I resolved to continue it in a higher potency. Gave Glonoine $^{200}$, every four hours.

October 12th.—Patient no worse; remedy continued as before.

October 13th.—Headache very much improved, and the face and eyes less congested, but the rheumatoid pains in the wrist had extended to the elbow and were much complained of. Temp., 101.5; pulse, eighty-six; respiration, twenty. Small petechial spots, like flea-bites, were noticed on the forearm and wrist, and this led to an examination of the skin elsewhere. The patient now mentioned, for the first time, that he had had for some days similar spots upon the legs. The legs from the knee to the ankle were covered with numerous small ecchymoses of varying size, and in some places, where several had coalesced as large as a silver dime. The knee of the left leg was tender and stiff, and the whole leg was pervaded with a peculiar sense of weakness and numbness. The patient was very restless and apprehensive; felt drowsy but could not sleep; the bowels, which previously had been regular, were now for three days constipated, with bitter taste, dry tongue, sore gums, and inappetence.

The petechiæ, the rheumatic pains in the wrist and knee-joint, the sense of weakness and prostration, the constipated bowels, the symptoms of the buccal cavity, the continued slow fever, and the insomnia with drowsiness, seemed to point clearly to Phosphorus, which was given, bi-hourly, in the sixth trituration.

October 14th.—Most of the symptoms remain about the same, but the pulse is ninety-two and weak, and the temperature has risen to 102.2°. The petechiæ have increased in number and size and have spread to the thighs and back. A slight nose-bleed occurred during the night. He feels greatly prostrated, but is restless and anxious, and his sleep after midnight was disturbed by vivid dreams, in which he thought he was climbing a great mountain, carrying a heavy load. The urine was scant and dark. Not seeing any clear indication for a change, Phosphorus was continued until the 16th inst., in varying potency, third, twelfth, thirtieth, and two-hundredth; but the patient grew slowly and manifestly worse, especially the pains and prostration.

October 16th.—Temp., 102.4; pulse, ninety, weak and trembling; respiration, twenty, shallow, as if unable to draw a full breath. The ecchymoses had extended over the entire body and were accompanied by much itching. The pain in the joints very severe, making him extremely irritable and restless. During the night he had had a copious nose-bleed. The urine scanty, with coffee-ground sediment.

An error in the remedy used being now apparent, led to a further study of the case. The character of the pain so closely resembled that of Rhus toxicodendron that its pathogenesy was examined, developing the following correspondences:

Fear of death; fears he will be poisoned.

Vertigo, worse from turning or stooping, or when rising from lying.

Headache, rush of blood to the head, with throbbing; restless; face red.

Eyes red and inflamed.

Epistaxis of coagulated blood, worse at night.

Face fiery red; dark-red; with burning.

Food, especially bread, tastes bitter.

Tongue dry, red, cracked.

Hunger without appetite.

Urine diminished; discharges a few drops of blood-red urine.

Pulse accelerated, weak, faint, and soft; trembling or imperceptible.

Tearing and burning in the shoulder and arm.

Pains felt mostly in the knee.

Swelling and stiffness of the joints.

Rheumatoid pains in the limbs, with numbness and tingling.

Great debility, soreness, and stiffness.

Restlessness, must change position.

Great sleepiness, with sleeplessness until midnight.
Dreams of great exertion, as rowing, swimming, etc.
Intolerable itching of the skin, with a red rash all over.

Rhus venenata was given in the thirtieth potency. This was chosen in preference to Rhus toxicodendron because of the profound depression of the nervous system, and for the reason that this Rhus is said to exert a stronger influence upon the cuticle; but I had no expectation that it would do anything more than reduce the fever and relieve the rheumatoid pains. In this I was very happily mistaken, for while the pains and the fever abated at once, the ecchymoses also ceased to extend, began to change color like an old bruise, and disappeared within ten days. The nose-bleed did not recur after the Rhus was taken, the fever was all gone by the second day, and the wrist and knee supple and free from pain by the fourth. The patient was discharged on October 26th, cured.

### II. A Cinchona Case.

Mrs. L. M. B., a native of England, aged thirty-seven, resident in New York about nine years; brunette; large and fleshy; originally of a ruddy complexion, but now pale and anæmic; the mother of four children, and in her last confinement, about one year previous to the date here mentioned, lost an enormous amount of blood, so much so as to endanger her life, since which time she has been feeble and dispirited; her menses have always been rather free, and at times menorrhagic. The husband, who had formerly been a good workman, had for the past year and a half taken to drink, and the family had sunken into absolute poverty. The wife had endeavored to support herself and children by taking in coarse washing, and her system was much run down by overwork, insufficiency of food, and constant anxiety. To these influences was probably due the severity of the hæmorrhage at her last confinement. The child, unfortunately, lived until its tenth month, when it died of capillary bronchitis. The exhaustion caused by nursing this child, and her untoward surroundings, brought on a low fever, for which she received large doses of Sulphate of Quinine from a dispensary doctor. This was the condition of things when I first saw her, in March, 1881. Through a charitable organization I secured the removal of the family from the wretched room they occupied in a rear building on Eleventh Avenue, near Twenty-eighth Street, to much healthier and cleaner quarters on Twenty-fourth Street, near Ninth Avenue. Work was found for the husband, who promised to reform, and who did maintain tolerably decent habits for some months thereafter.

A study of the patient's condition led me to give Natrum muriaticum, both because she had been dosed heavily with Quinine, and on account of various systems which corresponded with its pathogenesy; but, although it was continued for two weeks, in varying potency, with a milk and beef-tea diet, I saw no benefit from it. In some ways the patient was better, but these changes could well be ascribed to her improved surroundings and dietary.

She had a fever every day, beginning late in the forenoon, without chill, continuing until evening, and passing off with a copious sweat which lasted until near morning. The fever would vary day by day as to the hour of commencement, sometimes as early as ten o'clock, or as late as one o'clock, but never the same.

During the fever she was stupid, and could not be depended upon to describe her sensations. In the morning she had a bursting headache, and the congestion to the head apparently continued all day; but as soon as the perspiration set in all the untoward symptoms disappeared, she became lively and bright, said she felt very well and free from pain, and drank milk frequently and greedily. I stuck to Natrum longer than I otherwise should on account of one symptom—fever blisters on the lips—but finally changed to Nux vomica. This, Ignatia, Rhus tox., and Lycopodium were given during the next (third) week of treatment. The symptoms varied considerably and I was making a rather hopeless stern-chase after them, and felt very much discouraged, when a new phase presented itself and altered the entire outlook. Her menses came on the sixteenth day of treatment and were profuse. The discharge was watery, and contained numerous dark coagula. On the twenty-first day, the menses continuing, and the patient being now very weak and apathetic, I was shocked to find that there had appeared spontaneously several ecchymoses on the left thigh about the size of a silver dollar, and smaller ones on the leg, foot, and along the lumbar region. Phosphorus $^{12}$ was given, bi-hourly.

Twenty-second day.—The ecchymoses have spread considerably, the old ones enlarging and many new ones forming. Her face is shrunken and livid, with eyes surrounded by heavy blue lines; sight dim and uncertain; noises in the ear, like distinct bells; very apathetic, and either does not reply at all to questions, or slowly, as if she did not fully comprehend; desires continually cold lemonade, and refuses milk and the beef-tea, which disagree, causing eructations; urine scanty, turbid, and with a red-brown sediment; diarrhœa of bloody mucus, scanty, infrequent, painless; she wants to be bolstered up in bed on account of oppression in the chest when lying down; skin cold, clammy, and greasy; temperature (axilla), 103.4° F.

In the presence of so grave a condition, I naturally hesitated as to the best course to pursue. Evidently Phosphorus was doing no good. Various remedies, which had seemed indicated —at least, they were not given thoughtlessly and without much study—had been given, nevertheless, without result. I had avoided China, which had several times been called to my mind by symptoms in the case, because she had been so recently deluged with it. However, I could not disguise from myself the many points of resemblance between this drug and the case before me, and on studying its pathogenesis carefully I became convinced that if any remedy was capable of saving my patient it was China, and China only. China has the following:

Indifference; apathy; ill humor.

Dislike to all mental or physical exertion.

Slow train of ideas.

Intense throbbing headache—after loss of blood.

Sight dim and faint.

Fine ringing in ears.

Hardness of hearing; humming in ears.

Nose-bleed; ringing in ears; face pale.

Face pale, hollow, or livid; blue around the eyes; hippocratic.

Longs for sour, cooling things.

Violent thirst for cold drinks.

Sour eructations after milk.

Heartburn after milk.

Hæmatemesis; weak, pale, cold.

Stools: bloody, painless.

Urine: turbid, scanty; depositing brick-dust sediment.

Uterine hæmorrhages, ringing in ears, fainting, cold, loss of sight; discharge of dark clots.

Menses dark, coagulated; or pale and watery, with dark coagula.

Cannot breathe with head low.

Hæmoptysis.

Fever, long-lasting, and coming on at irregular intervals.

Sweat: partial, cold, or profuse; greasy.

Hæmorrhages from mouth, nose, or bowels; wants sour things.

Although the pathogenesis did not show ecchymoses on the skin or elsewhere, and I did not at that time know of the recorded poisonings in which purpura developed (Vepau), nevertheless, I determined, in view of the origin of the pathological state of the patient, resulting, as it did, from overlactation following excessive parturient hæmorrhage, and the remarkable coincidence in the concomitants, to give China, and in a high potency. I gave half-a-dozen pellets of Carroll Dunham's two hundredth, about noon, to be followed by a similar dose every four hours. Very little change was noted during the first twenty-four hours, except an improvement in the condition of the bowels; but on the twenty-third day the mental state was altered for the better in a marvelous degree and the fever temperature was only 100° F. All her apathy was gone, and she answered promptly and pleasantly all interrogatories. She took nourishment freely, had no perspiration at night, and slept quietly and soundly.

Twenty-fourth day.—No new ecchymoses have appeared since China was given, and many of the old ones are fading, changing to a mottled and greenish shade. She is now taking two quarts of milk daily, besides beef-extract. Bowels and kidneys acting normally. Temperature at noon, 99.4° F., but she is not conscious of any fever. She is very weak, but her mind is bright and her spirits high.

Twenty-seventh day.—She has continued to convalesce nicely. No fever to-day for the first time in two months. Appetite good, and functions all normal. The ecchymoses are fading slowly.

Thirty-second day.—She was up and moving about the room to-day. Has had an ounce of Speer's port wine, three times a day, with her meals, since the twenty-ninth. Is in all respects well except extremely weak. Has had no medicine since the twenty-eighth, except five drops of dialyzed iron in half an ounce of water at bedtime.

### III. A Secale Case.

On June 17th, 1881, I was called in to see a German woman, aged fifty-five, living on Twenty-ninth Street, opposite the old Hudson River Railway sheds. I had known the family for some time, as a son, a jeweler by trade, had a peculiar trouble of the heart. The old lady had not been ill for many years, but had for some months complained of a numbness of the left leg and foot, for which, however, she refused treatment, believing she could work it off. She was one of those dried-up little specimens, with a leathery skin, which we so often see among the poor class of German emigrants. The block on which they lived was notorious for its bad sanitary condition, and during the hot weather, which was now prevailing, funerals were a daily occurrence. She had been ill for several days, but refused to have medical attendance, as she had a great scorn for doctors. I was at the time attending that anomalous case of puerperal fever which I reported in the New York *Medical Times* for September, 1881, cured by Calcarea carbonica$^{200}$ (but which the editor printed after scratching out all reference to the potency), in the next house, and her daughter seeing me pass the door called me in to see her mother. The old lady refused to look at me or speak to me, but by using my eyes and from the report of the family I gathered the following facts in the case: She had had for two or three weeks a sensation on various parts of the skin, but most pronounced on the lower extremities, as of insects or vermin creeping about on her. She also complained of lack of sensation in her left foot and in both hands, which induced her to continually rub them with a piece of flannel. She had been taking some kind of German medicinal tea, the composition of which I did not learn. She evinced the greatest objection to lying in bed, and although very weak required constant supervision and persuasion to keep her there—and this when so exhausted that the attempt to get up only resulted in her sliding down upon the floor. Equally marked was her repugnance to being covered, and when I first saw her she lay in bed with nothing on but a short chemise and her native modesty. What attracted my attention first was the shrunken and anxious expression of her face, and next the peculiar appearance of her feet and legs. Both feet and legs up to the knee were covered with bruises, or what appeared to be such. These were much worse on the left side, where the toes were actually black. That this was not a mere local trouble was shown by the presence of ecchymoses upon the forearms and upon the buttocks. More alarming to the family, at least, was the emeto-catharasis. The vomiting and purging occurred simultaneously and involuntarily nearly hourly, but neither were very copious. The dejected matter was watery, nearly colorless, and preceded by colic and rumbling in the abdomen. Her skin was cold and clammy. She had a great thirst and was clamorous (or at least had been until her voice became so husky and weak as scarcely to be heard) for iced water, lemonade, beer, anything that was cold. She had had bleeding from the nose, but its character and frequency I could not learn. The urine was suppressed. Of course, there was never a doubt about the remedy; if in so desperate a case any drug could save it was Secale. Whatever the remedy did do it was not a "faith-cure," either on account of the doctor's mental attitude or the patient's, and I expected to find her dead on my return in the evening. Secale was given in the sixth trituration, dry on the tongue every ten minutes for an hour, and afterward half-hourly; a higher potency would have been given if I had had it with me. When I saw her four hours later, the vomiting had ceased, but the bowels remained about the same, except in frequency. The medicine was commenced at 2.30 P. M., and the diarrhœa ceased at about midnight. The reaction was followed by a slight fever, for which I gave the next day Aconite (this now I believe to have been a mistake), returning again to Secale in the evening, on account of her having had a diarrhœic stool. The purpura gradually faded, and quite disappeared in eight or nine days.

